# Global scenario of the RmtE pan-aminoglycoside-resistance mechanism: emergence of the *rmtE4* gene in South America associated with a hospital-related IncL plasmid

**DOI:** 10.1099/mgen.0.000946

**Published:** 2023-03-24

**Authors:** Jose F. Delgado-Blas, Cristina M. Ovejero, Sophia David, Carlos Serna, Mario Pulido-Vadillo, Natalia Montero, David M. Aanensen, Lorena Abadia-Patiño, Bruno Gonzalez-Zorn

**Affiliations:** ^1^​ Antimicrobial Resistance Unit (ARU), Animal Health Department, Faculty of Veterinary Medicine and VISAVET Health Surveillance Centre, Complutense University of Madrid (UCM), Madrid, Spain; ^2^​ Centre for Genomic Pathogen Surveillance (CGPS), Big Data Institute, Li Ka Shing Centre for Health Information and Discovery, University of Oxford, Oxford, UK; ^3^​ Bacterial Resistance Laboratory, Department of Biomedicine, Institute for Research in Biomedicine and Applied Sciences, University of Oriente (IIBCAUDO), Cumaná, Venezuela

**Keywords:** bacterial genomics, gene evolution, pan-aminoglycoside resistance, plasmid epidemiology, RmtE 16S rRNA methyltransferase

## Abstract

Antimicrobial resistance (AMR) mechanisms, especially those conferring resistance to critically important antibiotics, are a great concern for public health. 16S rRNA methyltransferases (16S-RMTases) abolish the effectiveness of most clinically used aminoglycosides, but some of them are considered sporadic, such as RmtE. The main goals of this work were the genomic analysis of bacteria producing 16S-RMTases from a ‘One Health’ perspective in Venezuela, and the study of the epidemiological and evolutionary scenario of RmtE variants and their related mobile genetic elements (MGEs) worldwide. A total of 21 samples were collected in 2014 from different animal and environmental sources in the Cumaná region (Venezuela). Highly aminoglycoside-resistant *Enterobacteriaceae* isolates were selected, identified and screened for 16S-RMTase genes. Illumina and Nanopore whole-genome sequencing data were combined to obtain hybrid assemblies and analyse their sequence type, resistome, plasmidome and pan-genome. Genomic collections of *rmtE* variants and their associated MGEs were generated to perform epidemiological and phylogenetic analyses. A single 16S-RMTase, the novel RmtE4, was identified in five *Klebsiella* isolates from wastewater samples of Cumaná. This variant possessed three amino acid modifications with respect to RmtE1–3 (Asn152Asp, Val216Ile and Lys267Ile), representing the most genetic distant among all known and novel variants described in this work, and the second most prevalent. *rmtE* variants were globally spread, and their geographical distribution was determined by the associated MGEs and the carrying bacterial species. Thus, *rmtE4* was found to be confined to *Klebsiella* isolates from South America, where it was closely related to IS*Vsa3* and an uncommon IncL plasmid related with hospital environments. This work uncovered the global scenario of RmtE and the existence of RmtE4, which could potentially emerge from South America. Surveillance and control measures should be developed based on these findings in order to prevent the dissemination of this AMR mechanism and preserve public health worldwide.

## Data Summary

All raw and assembled sequence data from this study have been deposited in the European Nucleotide Archive (ENA) at EMBL-EBI (https://www.ebi.ac.uk/ena) under the project PRJEB50384, and properly specified within the paper and its Supplementary Data files. Accession numbers for Illumina raw sequence data are indicated in Table S1 (available with the online version of this article). Accession numbers for Nanopore raw sequence data and Illumina–Nanopore hybrid assemblies are indicated in Tables S2 and S3, respectively. The sequence data from public repositories included in this work are available in Tables S4 and S5. Antibiotic resistance data for isolates are indicated in Table S6. Graphical visualizations of geographical/temporal data and phylogenetic trees of genes, genetic regions and plasmids included in this work are available from Microreact via specified web links: https://microreact.org/project/2RSzobARr3Bh7EntcfagKr-rmtevariantsworldwide, https://microreact.org/project/233bak1YcBTBCS7ePEtBiN-rmtevariantsgeneticenvironmentworldwide and https://microreact.org/project/2S69VARrPQAJSaESCg266D-inclcgsnptreeworldwide. All other relevant data are available from the corresponding author.

**Impact Statement**

16S rRNA methyltransferases (16S-RMTases) confer high-level resistance to most clinically relevant aminoglycosides, a critically important group of antibiotics for the treatment of complicated infections, including the last-resort aminoglycoside plazomicin. The epidemiology and dissemination of some clinically related and worldwide-spread 16S-RMTases, especially ArmA and RmtB, are largely understood. However, most 16S-RMTases are considered sporadic due to their low detection in clinical samples, such as RmtE. This work determined that RmtE is more disseminated than expected and encompasses multiple variants distributed globally. Genomic analysis of bacterial isolates from a ‘One Health’ perspective led to the identification of RmtE4 and its genetic environment in South America. This methodology allowed the early detection of a potential high-risk resistance gene and the establishment of further targeted surveillance and control measures to preclude the emergence of these genes in other pathogenic bacteria, the dissemination from the environment to other ecological niches, especially to human clinical settings, and the eventual expansion from a regional to a global level. Furthermore, the analysis of comprehensive genomic repositories revealed the genetic and bacterial elements associated with different *rmtE* variants. These large-scale studies are fundamental to understand the actual epidemiological scenario and the evolutionary dynamics of underestimated high-risk resistance genes.

## Introduction

Aminoglycosides are broad-spectrum antibiotics that have allowed the treatment of critical bacterial infections for the last 80 years [1]. They are unique among other antibiotics that disrupt protein synthesis processes, such as tetracyclines or macrolides, since these are bacteriostatic compounds, while aminoglycosides possess a bactericidal effect [2]. Specifically, aminoglycosides interact with the A-site of the 16S rRNA of the 30S ribosomal subunit, modifying its conformation and causing the generation of aberrant proteins whose abnormal functionality leads to the death of the bacterial cells [3]. Aminoglycosides can be classified in four basic groups according to chemical structure. The majority of most clinically relevant aminoglycosides, such as gentamicin and kanamycin, belong to the 4,6-disubstituted 2-deoxystreptamine (4,6-DOS) group [4]. In fact, the emergence and spread of multi-drug resistant (MDR) bacteria to other classes of antibiotics have positioned aminoglycosides as high priority compounds among critically important antibiotics for human medicine in recent times [5].

The most prevalent aminoglycoside-resistance mechanism is that involving the diverse aminoglycoside-modifying enzymes, but they display a limited spectrum of action [6]. The most concerning aminoglycoside-resistance mechanism for public health is that involving the acquired 16S rRNA methyltransferases (16S-RMTases), since they confer high-level resistance to all the clinically relevant 4,6-DOS aminoglycosides, even the last-resort aminoglycoside plazomicin, which is able to avoid the action of aminoglycoside-modifying enzymes [7]. This is due to the mechanism of action of the 16S-RMTases, consisting of the addition of a methyl group to specific residues of the 16S rRNA of the 30S ribosomal subunit and, therefore, blocking the interaction of aminoglycosides with their cellular target [8]. The genes encoding most of them are associated with various mobile genetic elements (MGEs), together with other resistance genes to other clinically relevant antibiotics, such as carbapenemase genes, leading to the emergence and spread of MDR pathogenic bacteria [9]. To date, a total of 10 different 16S-RMTases have been described, including ArmA, RmtA–RmtH and NpmA–NpmB; ArmA and RmtB being the most prevalent ones worldwide [10, 11]. However, most of the epidemiological studies are focused on clinical isolates from human samples, where ArmA- and RmtB-producing bacteria are predominant [10].

Other 16S-RMTases, such as RmtE, are considered sporadic due to their very low prevalence and are restricted to specific geographical regions. The first RmtE variant described, RmtE1, was originally identified in 11 *Escherichia coli* isolates from a cattle farm in the USA in 2010, associated with an untyped plasmid [12]. Afterwards, this variant was detected in two *E. coli* isolates obtained from clinical human samples in the USA, encoded in both an IncA/C plasmid and the bacterial chromosome and linked to the β-lactamase CMY-2 [13, 14]. Outside the USA, RmtE1 has only been identified in hospital samples from Myanmar and India, specifically in four *Pseudomonas aeruginosa *, five *Enterobacter xiangfangensis* and four *E. coli* isolates, in which it was associated with plasmid structures and co-expressed together with other relevant resistance proteins such as NDM-1 and CTX-M-15 [15–17]. The RmtE2 variant, with a single amino acid modification compared with RmtE1, was only described in two *E. coli* isolates from two diseased pigs in China in 2015, encoded in an IncI1 plasmid [18]. The other known variant, RmtE3, was recently described in two clinical *Acinetobacter baumannii* isolates from the United Kingdom and Venezuela, displaying one amino acid change with respect to RmtE2, but its genomic location remains undetermined [19].

The dissemination and epidemiological situation of the different acquired 16S-RMTase genes is very heterogeneous and depends to a great extent on the MGEs in which they are integrated, the other resistance genes with which they are associated, and the bacterial species and clones that carry them [20]. Some high-prevalence 16S-RMTases in human clinical and production animal isolates have been extensively studied [10]. However, the epidemiological situation of other 16S-RMTases with an apparent low prevalence, such as RmtE, remains largely unknown, especially in the community and environmental contexts, posing a high risk for the emergence and spread of clinical MDR bacteria. Thus, the goal of this work was the genomic study of *rmtE*-carrying bacteria from different animal and environmental sources in the Cumaná region of Venezuela, establishing the actual epidemiological scenario of this resistance mechanism, their known and novel variants, and the genetic platforms involved in their mobilization and dissemination worldwide.

## Methods

### Sampling and bacterial isolation

**Fig. 1.**
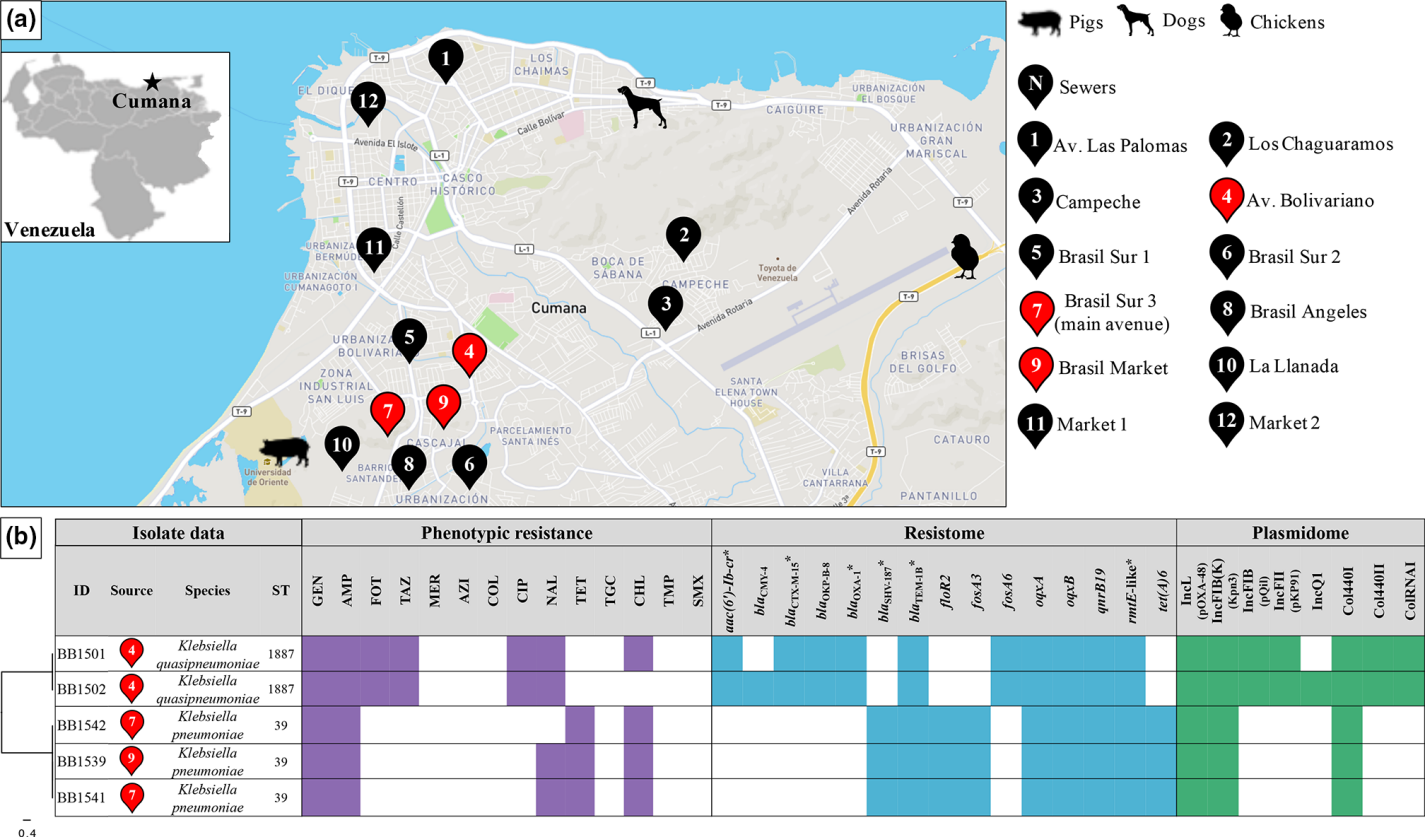
Geographical locations and sources of the samples from the Cumaná region (Venezuela) and data of the pan-aminoglycoside-resistant *Klebsiella* isolates recovered from the samples. (a) Inset map of Venezuela showing the location of Cumaná (black star). Amplified map of the Cumaná region displaying the location and source of the samples (see the key on the right). The sewers from where pan-aminoglycoside-resistant isolates were recovered are highlighted by red markers. (b) Core-genome SNP-tree of pan-aminoglycoside-resistant *Klebsiella* isolates (scale bar: substitutions per site), indicating the sewers according to the symbols displayed in (a), the *Klebsiella* species to which they belong and the STs. Phenotypic resistance to different antibiotics, assessed by MIC and according with the European Committee on Antimicrobial Susceptibility Testing (EUCAST) clinical breakpoints, is indicated by colour, whereas susceptibility is indicated by white. The total resistance gene content (resistome) and the total plasmid content (plasmidome) in terms of incompatibility groups are indicated by colours: presence by colour and absence by white. Antibiotics tested: GEN (gentamicin), AMP (ampicillin), FOT (cefotaxime), TAZ (ceftazidime), MER (meropenem), AZI (azithromycin), COL (colistin), CIP (ciprofloxacin), NAL (nalidixic acid), TET (tetracycline), TGC (tigecycline), CHL (chloramphenicol), TMP (trimethoprim) and SMX (sulfamethoxazole). Antimicrobial-resistance genes integrated in the IncL plasmid are denoted by asterisks.

A total of 21 samples were collected between May and July of 2014 from different hosts/environments in the Cumaná region (Venezuela): 3 canine faecal samples from stray dogs, 3 swine faecal samples from domestic pigs, 8 poultry faecal samples from a broiler farm and 7 wastewater samples from community sewers distributed across the region (Fig. 1a). All samples were homogenized in 9 ml 0.9 % NaCl solution (1 ml liquid samples and 1 g solid samples) and 100 µl was plated onto MacConkey agar (Oxoid) supplemented with 200 mg gentamicin l^−1^ plus 200 mg tobramycin l^−1^ (Sigma-Aldrich), in order to select highly aminoglycoside-resistant *Enterobacteriaceae* isolates. One bacterial colony of each different morphological type was retrieved from the aforementioned plates and stored for posterior analyses.

### Bacterial species identification, antimicrobial-susceptibility testing and 16S-RMTase gene detection

The bacterial species of the collected isolates were determined by MALDI-TOF MS at the VISAVET Health Surveillance Centre (Madrid, Spain), using a Bruker Daltonics UltrafleXtreme MALDI-TOF/TOF instrument and the Biotyper Real-Time Classification software version 3.1 with the MALDI Biotyper database (Bruker Daltonics). Minimum inhibitory concentration (MIC) values for *Enterobacteriaceae* isolates to different antimicrobial classes was evaluated by the broth microdilution method using commercial Sensititre EUVSEC plates (Trek Diagnostics), following the manufacturer’s specifications. The results were interpreted according to the European Committee on Antimicrobial Susceptibility Testing (EUCAST) guidelines [21]. PCRs to screen for the presence of all known 16S-RMTase genes (*armA*, *rmtA–rmtH* and *npmA*) were performed as previously described [22]. Further in-house broth microdilution assays were performed to determine the endpoint MIC values of 16S-RMTase-producing isolates for multiple aminoglycoside compounds (Sigma-Aldrich), including gentamicin, amikacin, kanamycin, neomycin, apramycin, streptomycin and the last-resort aminoglycoside plazomicin.

### Illumina and Nanopore whole-genome sequencing (WGS) and preliminary analyses

WGS of 16S-RMTase-producing *Enterobacteriaceae* isolates was carried out at the Instituto Tecnológico Agrario de Castilla y León (ITACYL) (Spain). A Nextera XT kit was used to obtain 300 bp paired-end libraries and sequencing was performed on a MiSeq platform (Illumina). Short-read sequences were processed for subsequent analysis by checking their sequencing quality with FastQC version 0.11.8 [23] and trimming low-quality end nucleotides using Trimmomatic version 0.39 [24], obtaining a mean of 47.8-fold coverage (minimum 18.6-fold, maximum 76.4-fold). Raw Illumina sequence data were deposited in the European Nucleotide Archive (ENA) (https://www.ebi.ac.uk/ena) under the project PRJEB50384, and individual accession numbers, metadata and quality metrics are indicated in Table S1.

For long-read WGS, total DNA extraction and purification were performed with a Wizard genomic DNA purification kit (Promega), following the Isolating Genomic DNA from Gram Negative Bacteria protocol. Subsequently, DNA quality and concentration were assessed by NanoDrop (Thermo Fisher) and Qubit (Invitrogen) devices. Genomic libraries were prepared according with the 1D Native barcoding genomic DNA protocol, using the EXP-NBD104 and SQK-LSK109 kits (Oxford Nanopore Technologies), and the sequencing run was carried out in a FLO-MIN106 flow cell with the MinION device (Oxford Nanopore Technologies). Downstream analysis was performed as follows: sequencing reads were basecalled with MinKNOW software (Oxford Nanopore Technologies), the demultiplexing process was developed via the Fastq Barcoding workflow of the Epi2Me interface (Metrichor), and trimming of adaptors and barcodes was completed by Porechop version 0.2.3 [25], obtaining a mean of 231.1-fold coverage (minimum 161.5-fold, maximum 306.2-fold). Raw Nanopore sequence data were deposited in the ENA under the project PRJEB50384, and individual accession numbers, metadata and quality metrics are indicated in Table S2.

### Long–short read hybrid assembly and genomic annotation

Illumina and Nanopore WGS data were combined to obtain hybrid assemblies using Unicycler version 0.4.7 [26] and applying default parameters. The quality of the assemblies was assessed with quast version 5.0.2 [27], displaying N50 values ranging between 5 300 217 and 5 338 793 bp. Complete genomes were evaluated with Bandage version 0.8.1 [28] and annotated with Prokka version 1.14.5 [29]. Plasmids carrying 16S-RMTase genes were located and inspected using the National Center for Biotechnology Information (NCBI) blast+ version 2.9.0 [30] with the ResFinder [31] and PlasmidFinder [32] databases, in order to determine the resistance gene content and the plasmid incompatibility group, respectively. Subsequently, these plasmid structures were extracted independently from each annotated genome to perform further analysis. Hybrid assemblies were submitted to ENA under the project PRJEB50384, and genome accession numbers, metadata and quality metrics are indicated in Table S3.

### Sequence type (ST), resistome, plasmidome, virulence and pan-genome analyses

Trimmed short-reads from Illumina sequencing were analysed with Kraken version 2.0.8 [33], applying the reference library for bacterial taxonomy of the NCBI database [34], in order to confirm bacterial species. STs were determined from hybrid assemblies by multilocus sequence typing (MLST) using the MLSTcheck tool version 2.1.1706216 [35] and the allele scheme for *Klebsiella pneumoniae *. Total resistance gene content and plasmid composition according to plasmid incompatibility groups were established following two different methodologies: using Illumina trimmed short-reads with ariba version 2.14.6 [36] and using Unicycler hybrid assemblies with ABRicate version 1.0.1 [37], applying the ResFinder and PlasmidFinder databases in both cases. Hybrid assemblies were also analysed with Kleborate version 0.3.0+ [38] to determine the presence of key actors associated with hypervirulence profiles.

The pan-genome analysis of all 16S-RMTase-producing *Klebsiella* spp. isolates was conducted with Roary version 3.13.0 [39], using the annotated hybrid assemblies from Prokka, to determine the gene clusters belonging either to core- or accessory-genome. The SNPs between gene clusters encompassed in the core-genome were extracted by the SNP-sites tool version 2.5.1 [40], and they were applied to generate a core-genome SNP-tree using RAxML version 8.2.8 [41] with 100 bootstrap iterations. The tree was visualized and edited with FigTree version 1.4.3 [42], as well as the tree based on the presence/absence of accessory-gene clusters obtained from the Roary pipeline.

### Collection and genetic analysis of *rmtE* variants and their associated MGEs worldwide

A collection of *rmtE* genetic sequences was generated by querying with NCBI blast sequences displaying >90 % nucleotide similarity and >90 % reference coverage from the NCBI database, and by searching and retrieving genomes containing the gene tag ‘*rmtE*’ included in the NCBI National Database of Antibiotic Resistant Organisms (NDARO) via the NCBI Pathogen Detection Isolates Browser (https://www.ncbi.nlm.nih.gov/pathogens/antimicrobial-resistance/) (databases were accessed in February 2021). A total of 124 assemblies containing *rmtE* and associated with minimum standardized metadata (sampling location at country level, collection date at year level and isolation source at human/animal/environmental level) were included in the *rmtE* collection (one isolate harboured two variants): 9 from the NCBI database, 111 from the NDARO and the 5 *rmtE*-carrying isolates from this work (Table S4). The assemblies were screened by ABRicate with ResFinder and PlasmidFinder databases in order to determine the total resistance gene content and plasmid incompatibility groups, and the contig where *rmtE* was integrated in each genome assembly was extracted and annotated via Prokka. Afterwards, the *rmtE* genetic sequence was extracted from each contig and all were aligned with the global alignment tool of Geneious version 8.1.9 [43], which also was used to align the RmtE amino acid sequences obtained from the translation of nucleotide sequences and check the amino acid changes. A tree was reconstructed from the nucleotide alignment using the Geneious Tree Builder, identifying all *rmtE* variants based on pairwise distances. The resulting tree and the metadata of the isolates from which the sequences were extracted were combined to generate a spatio-temporal visualization of *rmtE* variants with Microreact [44]. STs of *K. pneumoniae* isolates from the collection carrying the same *rmtE* variant to that identified in the isolates from this work were established by MLSTcheck tool, as previously indicated.

Complete annotated contigs containing *rmtE* were aligned with Progressive Mauve version 2.3.1 [45] in order to establish the common genetic modules shared by different contigs. Based on this alignment, the genetic environment of *rmtE* was extracted from each contig, including the upstream and downstream regions and the MGEs flanking the gene, when possible. Subsequently, *rmtE* genetic environments were aligned with ClustalW version 1.74 [46] and the resulting alignment was used to generate a tree with Geneious Tree Builder applying a bootstrapping of 1 000 replicates. A representative annotated sequence from each distinct *rmtE* genetic environment was manually curated according to the NCBI, ResFinder and ISFinder [47] databases and aligned in a pairwise comparison using EasyFig version 2.2.2 [48]. Lastly, a tanglegram linking the RmtE variant tree with the *rmtE* genetic environment tree was constructed with R version 3.6.1 [49] and RStudio version 1.2.1335 [50] using dendextend package version 1.15.1 [51] and ggtree package version 2.4.2 [52].

### Conjugation assays of *rmtE4*-carrying IncL plasmids and aminoglycoside-susceptibility testing of transconjugants

Conjugation assays were performed with one representative isolate of the two *rmtE4*-carrying *Klebsiella* species (* K. pneumoniae* BB1539 and *Klebsiella quasipneumoniae* BB1501) as donors and three laboratory strains belonging to different *Enterobacteriaceae* species (* K. pneumoniae* ATCC 700603, *E. coli* K12-MG1655 and *Salmonella enterica* subsp *. enterica* serovar Typhimurium ATCC 14028) as recipients. Recipient strains were transformed by electroporation with pBAD43 plasmid in order to use spectinomycin resistance as a selection marker for recipients and transconjugants. Donor and recipient cells were cultured with no antibiotics until donor cells reached late exponential phase (0.8–0.9 optical density at 600nm). Likewise, donor and recipient cells were co-cultured overnight. Conjugation rates were calculated as the number of transconjugants colonies (plates supplemented with 50 mg gentamicin l^−1^ plus 64 mg spectinomycin l^−1^) per number of donor colonies (plates supplemented with 50 mg gentamicin l^−1^). Phenotypic resistance levels of resulting transconjugants to multiple aminoglycoside compounds were assessed as described earlier in this paper.

### Collection and pan-genomic analysis of worldwide IncL plasmids related to those carrying *rmtE4* in Venezuela

The *repA* sequence of the *rmtE4*-carrying IncL plasmids identified in Venezuela was extracted and used as a reference to query the NCBI database (accessed in April 2021), retrieving all sequence entries with 100 % query cover and >99.8 % nucleotide identity (*n*=264). The collection and harmonization of metadata linked to the entries retrieved from the NCBI was performed with R and RStudio using the packages plyr version 1.8.6 [53] and data.table version 1.14.0 [54]. The minimum required metadata were: (i) circular topology, (ii) host bacterial species, (iii) sampling location at country level, (iv) collection date at year level and (v) isolation source at human/animal/environmental level. A collection of 174 complete plasmid sequences with all required metadata was generated, all of which were annotated with Prokka. All sequences were screened for the presence of three key genes of IncL/M type plasmids (*excA*, *traX* and *traY*), which are involved in the conjugation process and are used as molecular markers for phylogenetic classification [55]. Those sequences lacking one or more of these genes were removed from the collection and the sequence of the archetypical IncL plasmid R471 (GenBank accession no. KM406489) was included, as well as the sequences of the *rmtE4*-carrying IncL plasmids from Venezuela and one plasmid contig from the NCBI database sharing a 100 % nucleotide identity with the origin of replication of the Venezuelan plasmids (accession no. NCOO01000022), making a total collection of 169 IncL plasmid sequences (Table S5). The *excA*, *traX* and *traY* sequences were extracted from each plasmid and aligned, using the resulting alignment to build a phylogenetic tree for each gene with Geneious following the aforementioned procedure. Furthermore, all IncL plasmid sequences were inspected using ABRicate with a database of L-type replicon types in order to classify these plasmids into specific lineages, according to an L/M complex typing recently developed [56].

The pan-genome of IncL plasmids was established from the annotated sequence collection using the Roary pipeline as previously indicated, considering those genes present in at least 95 % of the sequences as core-gene clusters. The SNPs from all these core-genes were extracted with the SNP-sites tool and filtered with Gubbins version 1.4.10 [57] to remove hypervariable SNP positions. The remaining SNP alignment was used to generate a conserved core-genome SNP-tree using RAxML applying the aforementioned parameters. The resulting core-genome SNP-tree was visualized and edited with FigTree and combined with the metadata of the plasmids in Microreact to generate a spatio-temporal visualization of IncL type plasmids worldwide. In addition, the core-genome SNP-tree was also combined with the gene presence/absence matrix obtained from Roary in Phandango version 1.3.0 [58] to construct a graphical visualization of total IncL pan-genome. The IncL plasmid collection was subsequently screened via ABRicate to determine the resistance gene content, applying the ResFinder database as previously explained, and the results were integrated with the core-genome SNP-tree and visualized with Phandango. Then, those IncL plasmids belonging to the same cluster as *rmtE4*-carrying IncL plasmids (phylogenetic distance <10 SNPs) were aligned with Progressive Mauve to evaluate differences in the genetic content and/or organization. One representative sequence was selected for each different plasmid configuration and it was manually curated, according to the NCBI, ResFinder and ISFinder databases, to be included in a whole-length comparison generated with CGView Server version 2.0.2 [59]. Likewise, the variable region from each plasmid was identified, extracted and aligned in a pairwise comparison using EasyFig.

## Results and Discussion

### Novel RmtE4 variant confers pan-aminoglycoside resistance in wastewater *Klebsiella* spp. isolates from Venezuela

From all 21 samples obtained from different host/environments in the Cumaná region (Venezuela) (Fig. 1a), a total of 38 *Enterobacteriaceae* isolates were able to grow under high gentamicin and tobramycin concentrations (Table S6): 9 from canine faecal samples of stray dogs, 9 from swine faecal samples of domestic pigs, 11 from poultry faecal samples of a broiler farm and 9 from wastewater samples of community sewers distributed across the region. A sole 16S-RMTase gene was detected by PCR in 5 out of the 38 isolates (13.16 %): the uncommon *rmtE* gene. These isolates belonged to the genus *Klebsiella *, specifically three of them were identified as *K. pneumoniae* and the remaining two as *K. quasipneumoniae *, and they were recovered from three wastewater samples collected in sewers of the south-west area of the region (Fig. 1a, b), which possesses a high population density. Most of the 33 non-*rmtE* isolates were identified as *E. coli* (23 isolates) and *Enterobacter hormaechei* (8 isolates), and only 2 isolates belonged to species of the genus *Klebsiella *, which were obtained from one canine and one swine faecal sample. All non-*rmtE* isolates were resistant to the combination of gentamicin and tobramycin due to specific genes encoding aminoglycoside-modifying enzymes, specifically the acetyltransferase genes *aac(3)-IIa*, *aac(3)-IId* and *aac(3)-IVa*. These isolates exhibited a generally susceptible profile to other tested antibiotic classes, except for tetracycline, chloramphenicol, trimethoprim and sulfamethoxazole (Table S6). The genomes of all *rmtE*-carrying isolates were analysed by Illumina–Nanopore hybrid WGS. *K. pneumoniae* isolates came from two close sewers and were assigned to ST39, constituting a single clone with ~50 SNPs distance in the core-genome (5128/5172 core-genes, 99.1 % core-genome). This ST has been repeatedly described in the last few years as an emerging nosocomial MDR clone in Africa, Asia and Europe, frequently related with the production of carbapenemases and 16S-RMTases [60–62], which could increase the jeopardy of global dissemination of *rmtE4* from this region. *K. quasipneumoniae* isolates were recovered from the same sewer, which was different from the sewers where *K. pneumoniae* isolates were identified. They were assigned to ST1887 according to the *K. pneumoniae* MLST scheme, displaying a broad genetic distance of 589 SNPs in their reduced core-genome (5345/5615 core-genes, 95.2 % core-genome). This genetic distance could indicate either the intraspecies horizontal transfer of the gene or the regional diversification of the ST while maintaining *rmtE4*. Interestingly, ST1887 was first described in a clinical isolate from the adjacent Colombia the same year that the isolates of this work were collected [63], revealing the international spread of this ST in South America. Regarding the virulence of these isolates, no factors associated with hypervirulence profiles, including hypermucoviscosity genes, were identified, consistent with previous descriptions of low-virulence and high-resistance *Klebsiella* isolates [64].

The *rmtE* gene detected in all *Klebsiella* spp. isolates displayed a minimum of four nucleotide changes compared with *rmtE1-3* (454A>G, 510A>T, 646G>A and 800A>T), being the most genetically distant variant described to date. These changes caused three non-synonymous amino acid modifications with respect to RmtE1–3 (Asn152Asp, Val216Ile and Lys267Ile) (Fig. S1), resulting in a novel RmtE variant called RmtE4, following the nomenclature suggested by Doi *et al.* [65]. The gene *rmtE4* conferred high-level resistance to all 4,6-DOS aminoglycosides (MICs from 256 to >2048 mg l^−1^), including the last-resort antibiotic plazomicin, and it was located in an IncL type plasmid in all *Klebsiella* spp. isolates, which was absent in other bacterial genera (Table S6). This plasmid also harboured the gene *bla*_TEM-1B_ responsible for ampicillin resistance, among other β-lactamase genes depending on the species (Fig. 1b). Notably, due to the presence of *bla*_CTX-M-15_ in the IncL plasmid, *K. quasipneumoniae* isolates also exhibited high-level resistance to third-generation cephalosporins, linking two resistance mechanisms to last-resort antibiotics in the same genetic platform. In addition, *K. quasipneumoniae* isolates carried a broader plasmid content than *K. pneumoniae* isolates (Fig. 1b), which could be the cause of their larger total pan-genome and the more diverse genetic composition found in this species.

### RmtE variants are more diverse and globally spread than expected

**Fig. 2.**
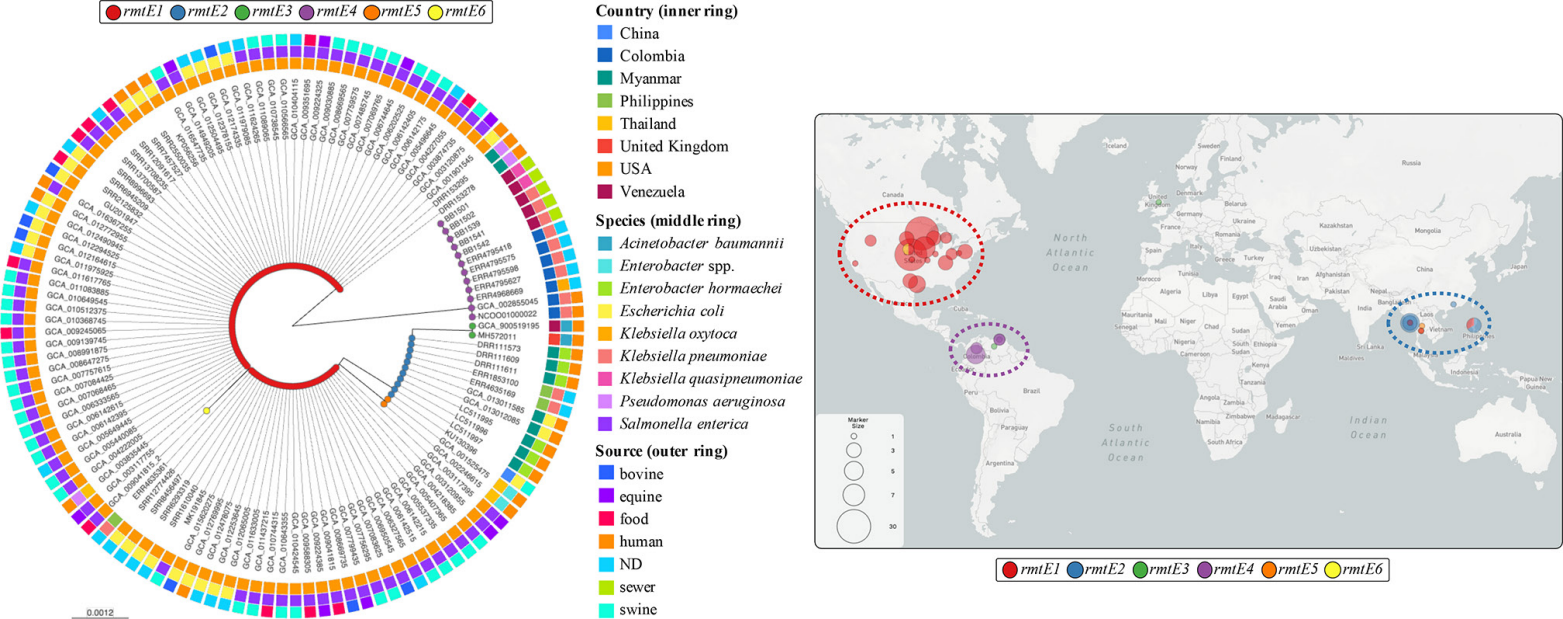
Phylogenetic tree of *rmtE* variants and their global distribution. SNP-tree of a total 125 *rmtE* genetic sequences collected from genomic repositories and our own WGS data (scale bar: substitutions per site) (left image). *rmtE* variants are indicated on the tips of the tree by coloured spots (see key at the top). The country of origin (inner ring), the carrying bacterial species (middle ring) and the source of isolation (outer ring) are indicated by different coloured squares (see key on the right of the tree). ND, Not determined. Map showing the global distribution of all 125 *rmtE*-carrying isolates (right image). *rmtE* variants are indicated by coloured circles (see key underneath the map). The size of the circles indicates the number of *rmtE*-carrying isolates (see key in the bottom left corner of the map). Dashed lines show the predominance of *rmtE* variants in each world region according with the colour of the variant (key underneath the map). Interactive visualizations of the tree and map are available from the Microreact website: https://microreact.org/project/2RSzobARr3Bh7EntcfagKr-rmtevariantsworldwide. Metadata of all the *rmtE*-carrying isolates are included in Table S4.

To investigate the actual scenario of RmtE, the NCBI database and the NDARO were inspected for *rmtE*-like containing genomes, retrieving a total of 124 assemblies with the minimum required metadata (see Methods). Interestingly, the comparison of all 125 *rmtE*-like genes (one isolate harboured two variants) and their resulting RmtE proteins allowed the identification of 6 different RmtE variants, including the already known RmtE1–3 and the RmtE4 identified in this work (Fig. 2). As expected, *rmtE1* was the most prevalent and widespread variant with 97 carrying isolates (77.6 %), exclusively associated with *Salmonella enterica* (70.1 %) and *E. coli* (25.8 %) from the USA, except for 4 isolates in South-East Asia, which belonged to other species (3 *P. aeruginosa* isolates from Myanmar and Thailand and 1 *K. pneumoniae* isolate from the Philippines) (Fig. 2; Microreact link – https://microreact.org/project/2RSzobARr3Bh7EntcfagKr-rmtevariantsworldwide). *rmtE1* was identified for what is believed to be the first time in isolates belonging to *Salmonella* and *Klebsiella* species, and, surprisingly, *rmtE1*-carrying *Salmonella enterica* accounted for almost three-quarters of all *rmtE1*-carrying isolates, which brings to light the successful expansion of this variant in *Enterobacteriaceae* species. Regarding *rmtE2*, its prevalence was notably lower compared with *rmtE1* (11 isolates, 8.8 %) and its distribution was restricted to South-East Asia and *Enterobacteriaceae* species (* Enterobacter hormaechei* in Myanmar, *K. pneumoniae* in the Philippines and *E. coli* in China). Only two isolates were associated with the variant *rmtE3*, both of them identified as *A. baumannii* and recovered from clinical human samples (Fig. 2), but the genetic and geographical distance (UK and Venezuela) discard a direct epidemiological link between them [19].

The novel variant *rmtE4* was identified in 12 isolates (9.6 %), including the 5 isolates characterized in the present work, making this variant the second most prevalent worldwide. Like other variants, the geographical distribution of *rmtE4* was confined to a particular region, comprising Colombia and Venezuela, where it was exclusively associated with members of the genus *Klebsiella *, mainly *K. pneumoniae *, but also *K. quasipneumoniae* and *Klebsiella oxytoca* (Fig. 2; Microreact link – https://microreact.org/project/2RSzobARr3Bh7EntcfagKr-rmtevariantsworldwide). In addition, two more unknown RmtE variants were uncovered in this work by computational analysis of genomic repositories, RmtE5 and RmtE6. The *rmtE5* variant was found in two *Enterobacter* spp. isolates recovered from clinical human samples in Thailand, showing a single nucleotide change compared with *rmtE1* (142G>A) responsible for a non-synonymous amino acid modification (Glu48Lys) (Fig. S1). In the case of *rmtE6*, it was identified in only one *S. enterica* isolate from the USA, which also carried the *rmtE1* variant, exhibiting a single nucleotide change with respect to *rmtE1* (745A>G) that translated into one amino acid modification (Ile249Val) (Fig. S1). Despite that the capacity of aminoglycoside resistance attributable to Rmt5 and Rmt6 is not clear due to the lack of phenotypic resistance data associated with their carrying isolates, the predicted secondary structures and physico-chemical properties of the aforementioned amino acid changes are compatible with total enzymatic functionality. Future phenotypic studies are required to elucidate the potential pan-aminoglycoside resistance conferred by RmtE5 and RmtE6.

The present work uncovered that *rmtE*-carrying bacteria are not that scarce, and they are globally distributed, but not particularly associated with human clinical samples. This could be the reason why this 16S-RMTase gene is found less than others more prevalent in human isolates. Thus, comprehensive studies of novel/emerging resistance genes linked to animal and environmental sources are required to assess their potential risk to public health [66]. The close genetic relationship between all *rmtE* variants suggests a recent parallel evolution from a common ancestor, probably related to *rmtE1*, since this variant showed the highest prevalence and the shortest genetic distance compared with the others. This hypothesis could be supported by the fact that the novel *rmtE6* was found coexisting with *rmtE1* in the same bacterial cell, pointing at this variant as the origin of *rmtE6*.

### Spread of *rmtE* variants is linked to specific MGEs and bacterial species

**Fig. 3.**
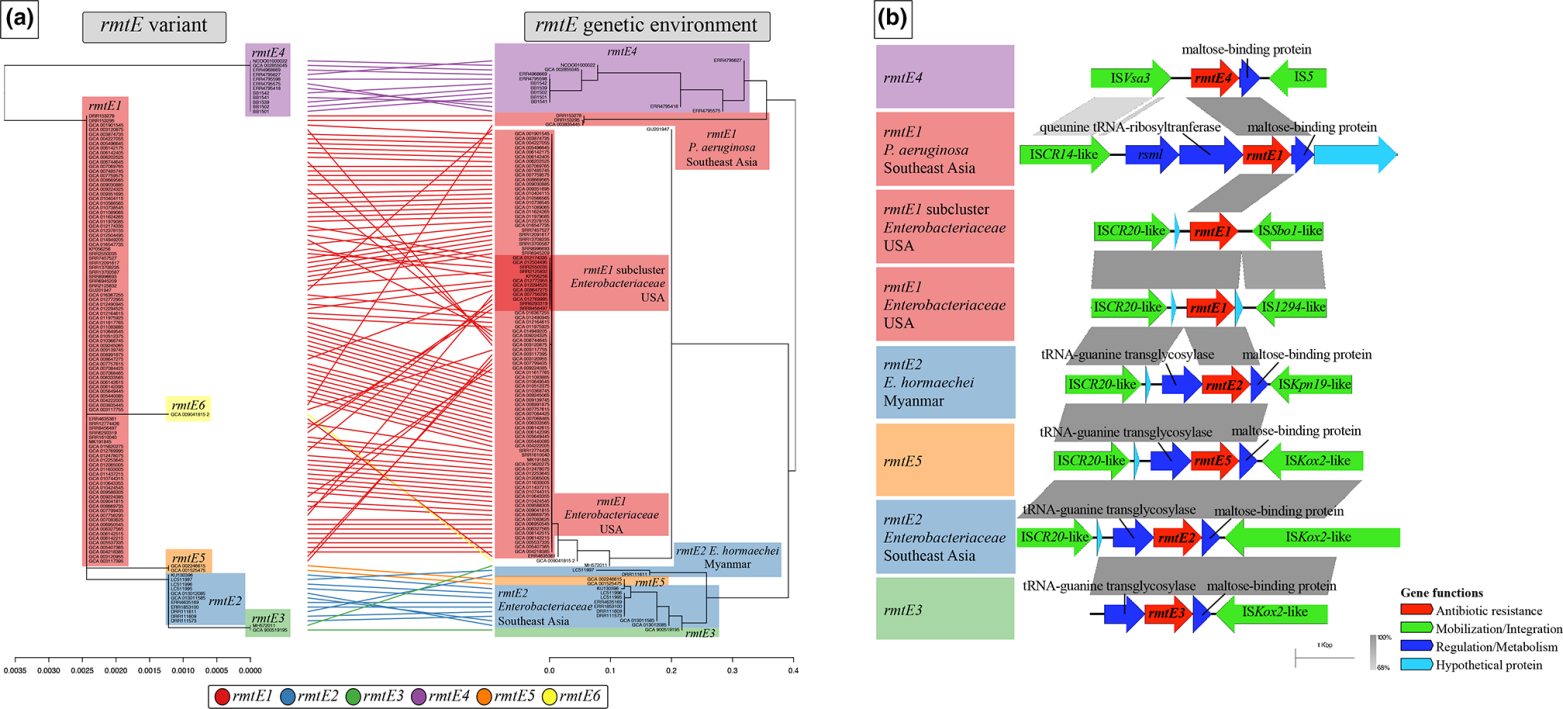
Association of *rmtE* variants and their flanking genetic environments. (a) Tanglegram linking the SNP-tree of *rmtE* genetic sequences with the total SNP-tree of their flanking genetic environments (from MGE to MGE when possible) (scale bars: substitutions per site). *rmtE* variants are indicated by coloured areas (see key at the bottom). The coloured areas of the genetic environments are coloured according with the variants and labelled regarding specific genetic clusters. Interactive visualizations of the tree and map of *rmtE*-flanking genetic environments are available from the Microreact website: https://microreact.org/project/233bak1YcBTBCS7ePEtBiN-rmtevariantsgeneticenvironmentworldwide. (b) Pairwise comparison of representative *rmtE*-flanking genetic environments. Each sequence is representative of one specific genetic cluster and it is coloured and labelled in accordance with (a). Curated annotations are represented by arrows, labelled with the gene name/predicted function and coloured depending on groups of genetic functions (see key at the bottom right). Pairwise nucleotide identity (%) is displayed by grey-coloured areas between representative sequences (according to the scale shown at the bottom right).

The *rmtE* variants were linked to distinctive genetic environments, including the flanking insertion sequences (ISs) and the plasmid types in which they were integrated, and these environments were determined by the carrying bacterial species and the geographical region (Fig. 3a). The *rmtE1* variant possessed a common IS*CR20*-like element in its upstream region and one of two highly related IS*91* elements in its downstream region, either IS*1294*-like or IS*Sbo1*-like, in all *Enterobacteriaceae* isolates from the USA (Fig. 3b; Microreact link – https://microreact.org/project/233bak1YcBTBCS7ePEtBiN-rmtevariantsgeneticenvironmentworldwide). Interestingly, all *S. enterica* isolates recovered from horse clinical samples in different locations of the country carried this *rmtE1*-flanking structure integrated in a common IncI1 plasmid, which also harboured *bla*_CMY-2_, but these bacteria belonged to different serotypes, pointing at an outbreak expansion at a local level [67] and plasmid dissemination at a national level. However, the closest upstream MGE found in *rmtE1*-carrying *P. aeruginosa* isolates from Myanmar and Thailand was an IS*CR14*-like element (Fig. 3b), suggesting the parallel emergence of *rmtE1* in *Enterobacteriaceae* isolates from North America and *P. aeruginosa* isolates from South-East Asia. Regarding *rmtE2*, all carrying isolates shared the same IS*CR20*-like element found in the upstream region of *rmtE1*-carrying *Enterobacteriaceae* isolates. However, the downstream region of *rmtE2* in *Enterobacter hormaechei* isolates from Myanmar was formed by either IS*Kox2* or IS*Kpn19* (Fig. 3b), which could indicate the risk of this species as a generator of novel *rmtE*–MGE associations. In fact, the *rmtE5* gene identified in this work was found in two *Enterobacter* spp. isolates from the neighbouring Thailand, integrated in the same structure as *rmtE2* (IS*CR20-rmtE5*-IS*Kox2*) (Fig. 3b). In addition, and despite the large geographical distance, the two *rmtE3*-carrying *A. baumannii* isolates from Venezuela and the UK also showed this identical IS*Kox2* downstream of the gene (Fig. 3b; Microreact link – https://microreact.org/project/233bak1YcBTBCS7ePEtBiN-rmtevariantsgeneticenvironmentworldwide). However, the upstream region of *rmtE3* could not be characterized due to the limited size of the contigs in which it was identified, as well as the genomic location of this variant [19]. The presence of IS*CR20*-like elements in the upstream region of *rmtE1*, *rmtE2* and *rmtE5*, and IS*Kox2* in the downstream region of *rmtE2*, *rmtE3* and *rmtE5*, revealed the essential role of this MGE in the genetic diversification and interspecies dissemination of this resistance mechanism at a global level. The *rmtE6*-contig only contained the coding sequencing of the gene, impeding the analysis of its flanking elements, although the coexistence of this gene with *rmtE1* could suggest that they shared a common genetic background.

The *rmtE4* variant not only was the most genetically distant, but also possessed the most distinct genetic environment among all *rmtE* variants (Fig. 3a). The adjacent upstream region contained one copy of IS*Vsa3*, an element known for its ability to mobilize and integrate other resistance genes to last-resort antibiotics [68, 69], which could also be responsible for the mobilization of *rmtE4* from its primary ancestor. The downstream region was flanked by an IS closely related with IS*Pst7* (91 % nucleotide identity) (Fig. 3b), an element of the IS*5* family that originated in the chromosome of *Pseudomonas stutzeri *. The IS*Vsa3-rmtE4*-IS*Pst7*-like structure was identical in all *rmt4*-carrying isolates and, therefore, it was exclusively associated with members of the genus *Klebsiella* from the region of Colombia and Venezuela (Microreact link: https://microreact.org/project/233bak1YcBTBCS7ePEtBiN-rmtevariantsgeneticenvironmentworldwide), where this structure was integrated in a particular IncL type plasmid in almost all the cases. The *rmtE4*-carrying *Klebsiella* isolates from Venezuela were confined to wastewater samples, an ecological niche where diverse genetic and bacterial elements converge and lead to novel gene–MGE–bacterium associations, especially in low- and middle-income countries (LMICs) [70]. The STs of the Colombian *rmtE4*-carrying *K. pneumoniae* isolates were different to those identified in the Venezuelan ones (ST37, ST258, ST280 and ST307), uncovering the international dissemination of *rmtE4* in multiple *K. pneumoniae* clones. Moreover, the *rmtE4*-carrying IncL plasmid was able to be transferred by conjugation from harbouring *Klebsiella* species to all *Enterobacteriaceae* species tested. As expected, conjugation rates were higher between *Klebsiella* species: 5.16×10^−4^ and 4.51×10^−4^ from *K. pneumoniae* and *K. quasipneumoniae* to *K. pneumoniae *, respectively. Nonetheless, conjugation rates were also high from these donor species to *E. coli* (5.65×10^−5^ and 6.51×10^−5^, respectively), and lower from *K. pneumoniae* to *S. enterica* Typhimurium (3.02×10^−7^). No transconjugants were obtained from *K. quasipneumoniae* to *S. enterica* Typhimurium. All transconjugants exhibited a high phenotypic resistance to all 4,6-DOS aminoglycosides (MICs from 128 to >2048 mg l^−1^), including plazomicin. All recipient strains belonging to different bacterial species increased the resistance levels to these antibiotic compounds by at least 64-fold (Table S6). These results confirmed the high transfer risk of IncL plasmid to other clinically relevant *Enterobacteriaceae* species, and the resulting transfer of the high-level resistance to most clinically used aminoglycosides conferred by RmtE4.

### *rmtE4* variant is associated with an uncommon IncL plasmid related to hospital environments in America

Of the 12 *rmtE4*-carrying *Klebsiella* isolates, the gene was integrated in a plasmid belonging to the IncL type in 10 of them: the 5 Venezuelan isolates identified in this work, 2 hospital isolates from the neighbouring Colombian department of Santander [71] and 3 isolates from Bogotá (Colombia) included in a genomic surveillance project (PRJEB29742). The remaining two isolates were also from Bogotá and belonged to ST307 but, regarding their plasmid content, they lacked IncL type plasmids. Due to the limited length of the *rmtE4*-containing contigs, the identification of the *rmtE4*-carrying plasmid in these isolates was not possible, but they both presented the identical IS*Vsa3-rmtE4*-IS*Pst7*-like structure shared by all *rmtE4*-carrying isolates, demonstrating the ability of this IS–gene association to be mobilized between different bacterial species and genomic locations. The predominant *rmtE4*-carrying IncL plasmid, called pRMTE4, possessed a *repA* sequence that shared 99.8 and 94.9 % nucleotide identity with the *repA* sequences of the archetypical IncL plasmids pOXA-48 (accession no. JN626286) and R471 (accession no. KM406489), respectively (Fig. S2A). To understand the genetic context and the epidemiological scenario of pRMTE4, a database of 169 IncL type plasmids sequenced worldwide was generated, all of them linked to minimum required metadata and presenting the key IncL/M conjugation genes *excA*, *traX* and *traY* (see Methods). When comparing the sequences of these three genes, which are used as phylogenetic markers [55], the cluster in which pRMTE4 was included was the most divergent of all IncL type plasmids, showing 98.8–98.9, 99.7–99.8 and 99.6 % nucleotide identity with pOXA-48 and R471 *excA*, *traX* and *traY* genes, respectively (Fig. S2B–D). This IncL cluster belonged to the L3 lineage according with the L/M complex typing recently described [56], whereas the pOXA-48 cluster and R471 corresponded to the L4 and L1 lineages, respectively.

The pan-genome analysis revealed the large and diverse genetic content of IncL plasmids, exhibiting a total of 494 gene clusters of which only 36 were considered core-genes (7.3 % core-genome, 95 % presence threshold). This genetic diversity was mainly due to pOXA-48 plasmids, since their core-genome was formed by 38 out of their 430 total genes (8.8 %), whereas the L3 lineage core-genome was much larger with 78 out of the 133 total genes (58.65 %) (Fig. S3). However, this contrast could be caused by the enormous overrepresentation of pOXA-48 plasmids in genomic repositories and, therefore, in the IncL plasmid database generated in this work (156/169, 92.3 % pOXA-48 plasmids). Among the genes conferring this large genetic variability in pOXA-48 plasmids were those related with antimicrobial resistance (AMR) (26 different resistance genes), but most of them only carried the carbapenemase gene that gives them their name, *bla*_OXA-48_ (Fig. S4). However, the L3 lineage showed a lower resistance gene diversity (15 different genes), which could also be due to the limited identification of this plasmid type comparing with pOXA-48, but all of them carried three resistance genes to different antibiotic groups at least (Fig. S4), favouring the expansion of this plasmid lineage in nosocomial environments.

**Fig. 4.**
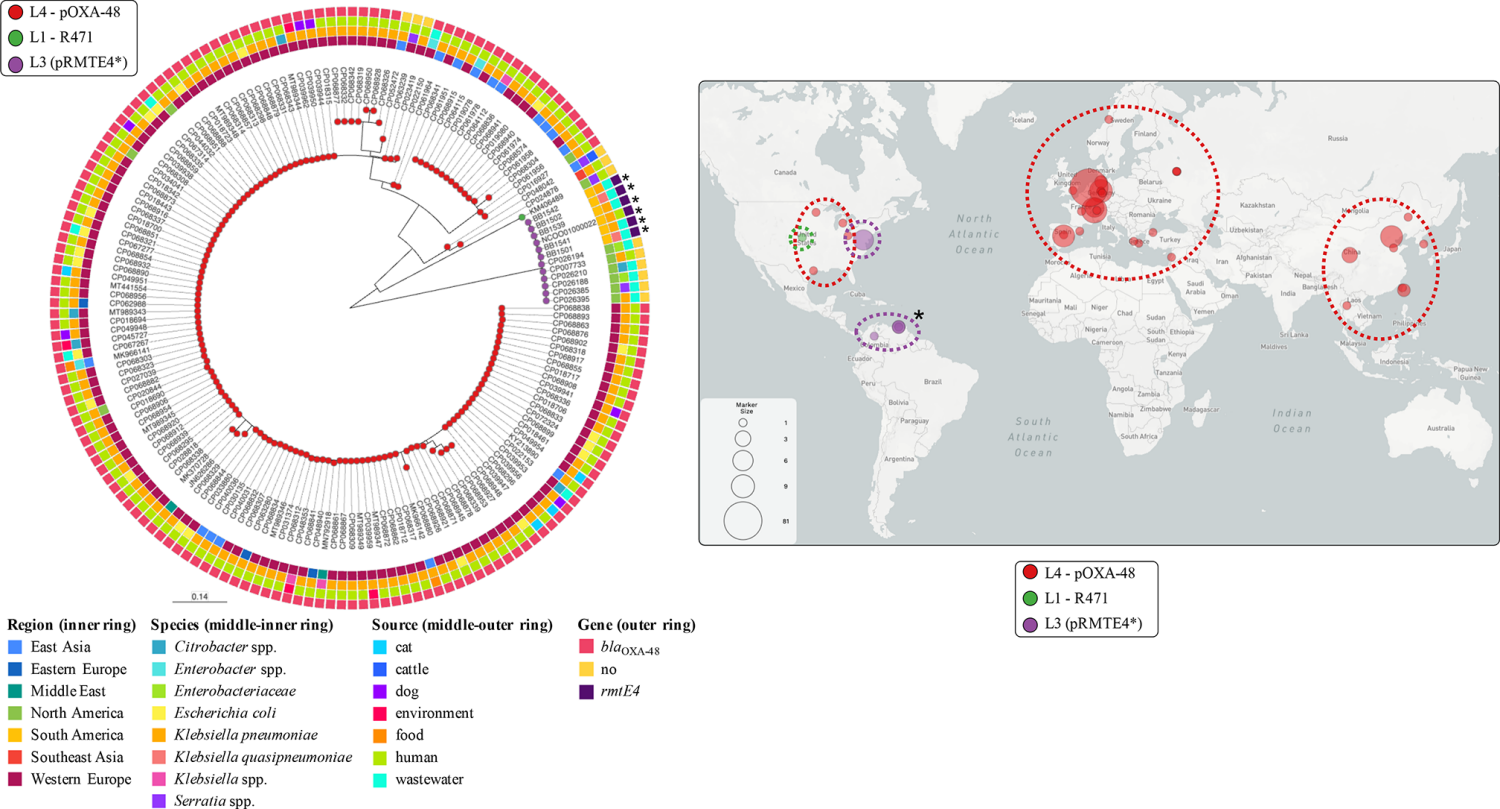
Phylogenetic tree of IncL type plasmids and their global distribution. Core-genome SNP-tree from non-recombinogenic regions of 169 IncL plasmids sequences collected from genomic repositories and our own WGS data (scale bar: substitutions per site) (left image). Specific IncL types are indicated on the tips of the tree by coloured spots (see key in the left top corner) and pRMTE4 plasmids are indicated by asterisks. The world region of origin (inner ring), the carrying bacterial species/genus (middle-inner ring), the source of isolation (middle-outer ring) and the presence/absence of *bla*_OXA-48_/*rmtE4* genes (outer ring) are indicated by different coloured squares (see key underneath the tree). Map showing the global distribution of all 169 IncL type plasmids (right image). Specific IncL types are indicated by coloured circles (see key underneath map). The size of the circles indicates the number of IncL-carrying isolates (see key in the bottom left corner of the map). Dashed lines show the predominance of specific IncL types in each world region according to the colour of the variant (key underneath map) and pRMTE4 plasmids are indicated by asterisks. Interactive visualizations of the tree and map are available from the Microreact website: https://microreact.org/project/2S69VARrPQAJSaESCg266D-inclcgsnptreeworldwide. Metadata of all IncL-carrying isolates are included in Table S5.

**Fig. 5.**
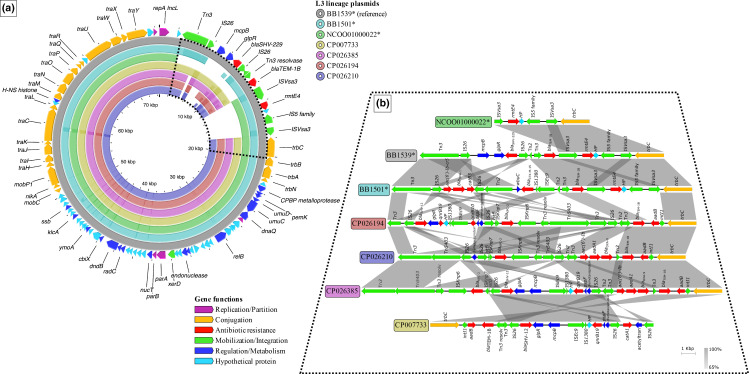
Genetic comparison of total length and variable regions of L3 lineage plasmids. (a) Total genetic comparison of representative L3 plasmids against the reference (BB1539). Each representative plasmid is displayed by a different colour (see key at the top right) and pRMTE4 plasmids are indicated by asterisks. Genetic regions shared with the reference are indicated by coloured blocks, whereas distinct regions are displayed by gaps. Darker-coloured areas within the shared regions indicate genetic elements in more than one copy in the same plasmid, and their level of identity compared with the reference plasmid. Curated annotations for the reference sequence (BB1539) are represented by arrows in the outer ring, labelled with the gene name and coloured depending on groups of genetic functions (see the key at the bottom right). The dashed black line frames the variable region of L3 plasmids. (b) Pairwise comparison of representative L3 plasmids. Each sequence is coloured and labelled according to (a). Curated annotations are represented by arrows, labelled with the gene name/predicted function and coloured depending on groups of genetic functions (the same as in a). Pairwise nucleotide identity (%) is displayed by grey-coloured areas between representative sequences (according to the scale shown at the bottom right).

In accordance with the genetic variation of the aforementioned conjugation genes, the genetic divergence resulting from core-genome SNPs displayed a clear distinction between IncL plasmid types, in terms of SNPs in non-recombinogenic core-genes. L3 lineage plasmids, including pRMTE4, presented a mean distance of 157 SNPs with respect to pOXA-48 plasmids and ~121 SNPs compared with R471, similar to the distance between pOXA-48 and R471 (~125 SNPs) (Fig. 4; Microreact link – https://microreact.org/project/2S69VARrPQAJSaESCg266D-inclcgsnptreeworldwide). The pOXA-48-like cluster showed a greater genetic diversity among its members, with a mean distance of 16 SNPs and a maximum of 112 SNPs, which could be caused again by the high prevalence of this plasmid type in *Enterobacteriacea* e isolates from clinical settings worldwide and, therefore, their overrepresentation in genomic repositories (Fig. 4). However, the 12 plasmids belonging to the L3 lineage were extremely conserved and geographically restricted, with only three SNPs in their core-genome and two non-synonymous changes in the conjugation gene *traJ* and the DNA repair gene *radC*, differentiating two subclusters. One subcluster comprised six plasmids from environmental *Enterobacteriaceae* isolates collected in a single hospital in Washington DC without the *rmtE4* gene [72], and the other subcluster included the six *rmtE4*-carrying pRMTE4 plasmids from Venezuela and Colombia exclusively related with *Klebsiella* species (Fig. 4; Microreact link – https://microreact.org/project/2S69VARrPQAJSaESCg266D-inclcgsnptreeworldwide). It should be pointed out that the amino acid modifications generated by the SNPs identified in *traJ* and *radC* did not cause any conformational change in the secondary structure of the proteins. When comparing the genetic content of all L3 lineage plasmids, the 78 core-genes of the plasmid backbone were highly conserved and encompassed genes related to replicative, conjugative, metabolic and regulatory functions (Fig. 5a). Regarding the accessory-genome, the vast majority of it encoded MGEs, namely ISs and transposons, and antimicrobial-resistance determinants (Fig. 5b). Some of these MGEs were shared by all members of the L3 lineage, especially the ubiquitous Tn*2*, Tn*3* and IS*26*, allowing the maintenance of closely related resistance genes in all plasmids, such as the β-lactamase *bla*_TEM-1B_, as described elsewhere [56]. In addition, these elements also generated mutable MGE-resistance gene modules that led to the divergence between subclusters, especially the association of multiple IS*26* copies with distinct *bla*_SHV_ and *bla*_OXA_ variants in accordance with the subcluster (*bla*_SHV-12_ and *bla*_OXA-9_ in some USA plasmids and the novel *bla*_SHV-229_ and *bla*_OXA-1_ in some South American plasmids) (Fig. 5b). However, some MGE-resistance gene modules were specifically related with each subcluster, including the worrisome carbapenemase gene *bla*_KPC-2_ associated with the typical IS*Kpn* elements in most USA hospital isolates and the novel 16S-RMTase variant *rmtE4* coupled with IS*Vsa3* in all South American plasmids (Fig. 5b). The close phylogenetic link between these plasmid subclusters pointed at a common plasmid ancestor. However, the distinct genetic content and the limited geographical distribution suggested a parallel evolution during which the South American subcluster acquired the *rmtE4* gene.

### Surveillance and control measures to preclude RmtE4 health impact

The comprehensive study of novel resistance genes from a ‘One and Global Health’ perspective is essential to understand their evolutionary and epidemiological dynamics and forecast their potential to reach human pathogenic bacteria and spread worldwide. The association of the *rmtE4* gene with a hospital-related IncL plasmid in clinical and environmental *Klebsiella* isolates entails a significant risk of emergence of this resistance determinant from South America to other regions. The identification of newly emerged/emerging AMR genes is the first step to develop successful surveillance strategies [73]. This is particularly important in LMICs, where there is a lack of surveillance and up-to-date information regarding AMR [74]. The environment and, specifically, wastewater surveillance have the ability to identify newly emerging or recently mobilized genes that have not yet become fixed in a microbial population. This would be especially valuable for guiding policy action and the investment of resources where they are most needed in a manner appropriate to the local situation. The genetic and phenotypic characterization of *rmtE4* is the basis to develop qPCR (quantitative real-time PCR) and phenotypic screening techniques that allow the quick detection of the carrying bacteria, which is key for prevention, intervention and control [75]. Hence, this gene can be included in qPCR panels for surveillance to monitor its epidemiological dynamics. Furthermore, clinically relevant resistance genes detected in wastewater are likely of exogenous origin, possibly derived from human antibiotic usage [76]. Thus, the usage reduction of aminoglycosides would help to control the potential spread of *rmtE4*-carrying bacteria, informing human and animal clinicians about other antibiotics that will be most effective at population-specific scales [74]. This control measure is especially relevant, since this gene is encoded in a conjugative plasmid, which increases the risk of transfer to pathogenic bacteria and emphasizes the need for further monitoring in order to prevent further spread of this gene in the environment [76]. The early detection of a resistant bacteria in wastewater could indicate towards potential dissemination within the community, allowing valuable time for appropriate preparation and response to be put into place. For example, the environmental conditions in sewers have been shown to potentially impact resistance-gene abundance, including temperature, metal pollutants and changes in composition of microbial communities [75]. Thus, the inspection and improvement of canalization and sewers in the Cumaná region could contribute to the control of *rmtE4* dissemination. Furthermore, the proper disposal of solid waste is required, especially in LMICs, including the disposal of unused and expired antimicrobials to prevent their unnecessary exposure to micro-organisms in the environment [77]. Decentralized systems in the removal and release of AMR genes and their carrying bacteria in aquatic environments should be evaluated, such as peracetic acid and algae ponds with high pH, which can be useful for their elimination in LMICs [78]. Likewise, the anaerobic–aerobic sequencing treatment of domestic wastewater has been demonstrated to provide the most reduction in AMR gene levels, including aminoglycoside-resistance genes [79].

In conclusion, the results from the present work are the foundation to develop and establish surveillance and control actions at a regional level in Cumaná in order to prevent the dissemination of the RmtE4 pan-aminoglycoside resistance mechanism at a global level.

## Supplementary Data

Supplementary material 1

Supplementary material 2
