# Supplementary material for: Global scenario of the RmtE pan-aminoglycoside-resistance mechanism: emergence of the rmtE4 gene in South America associated with a hospital-related IncL plasmid
Source: Microb Genom. 2023 Mar 24;9(3):mgen000946. doi: 10.1099/mgen.0.000946 (PMC10132066; doi:10.1099/mgen.0.000946)
Supplement: Supplementary material 1 [file mgen-9-946-s001.pdf]

Supplementary Figures

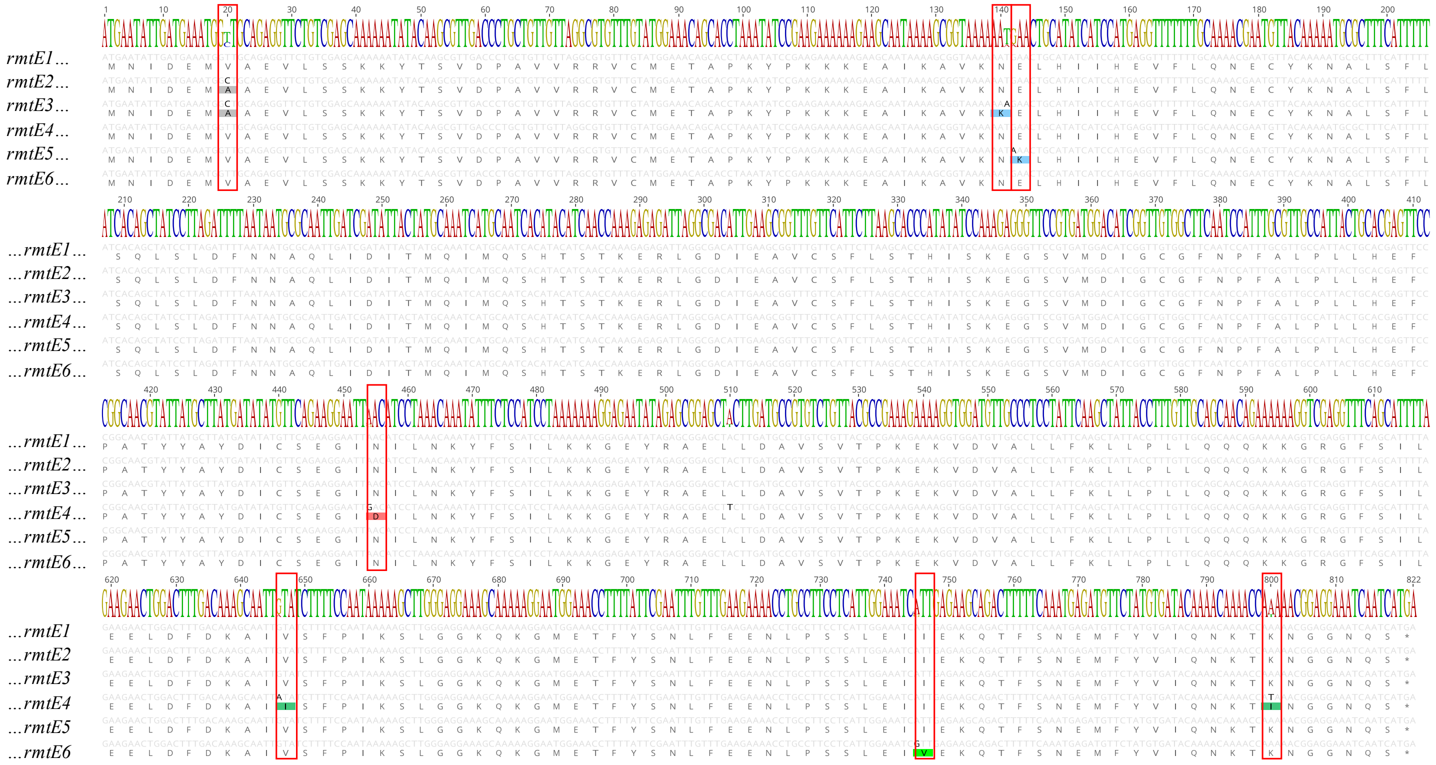

Supplementary Figure 1. Nucleotide comparison of *rmtE* variants. Nucleotide position is indicated on the top of the graph, followed by the consensus nucleotide sequence. Each nucleotide codon is translated to its related amino acid, and those causing non-synonymous modifications are highlighted and colored according with the amino acid. Aminoacidic positions with non-synonymous modifications are framed by a red rectangle. *rmtE1* reference: GU201947. *rmtE2* reference: KU130396. *rmtE3* reference: MH572011. *rmtE4* reference (from this work): BB1502. *rmtE5* reference (identified in this work): GCA_002246615 (assembly). *rmtE6* reference (identified in this work): GCA_009041815 (assembly).

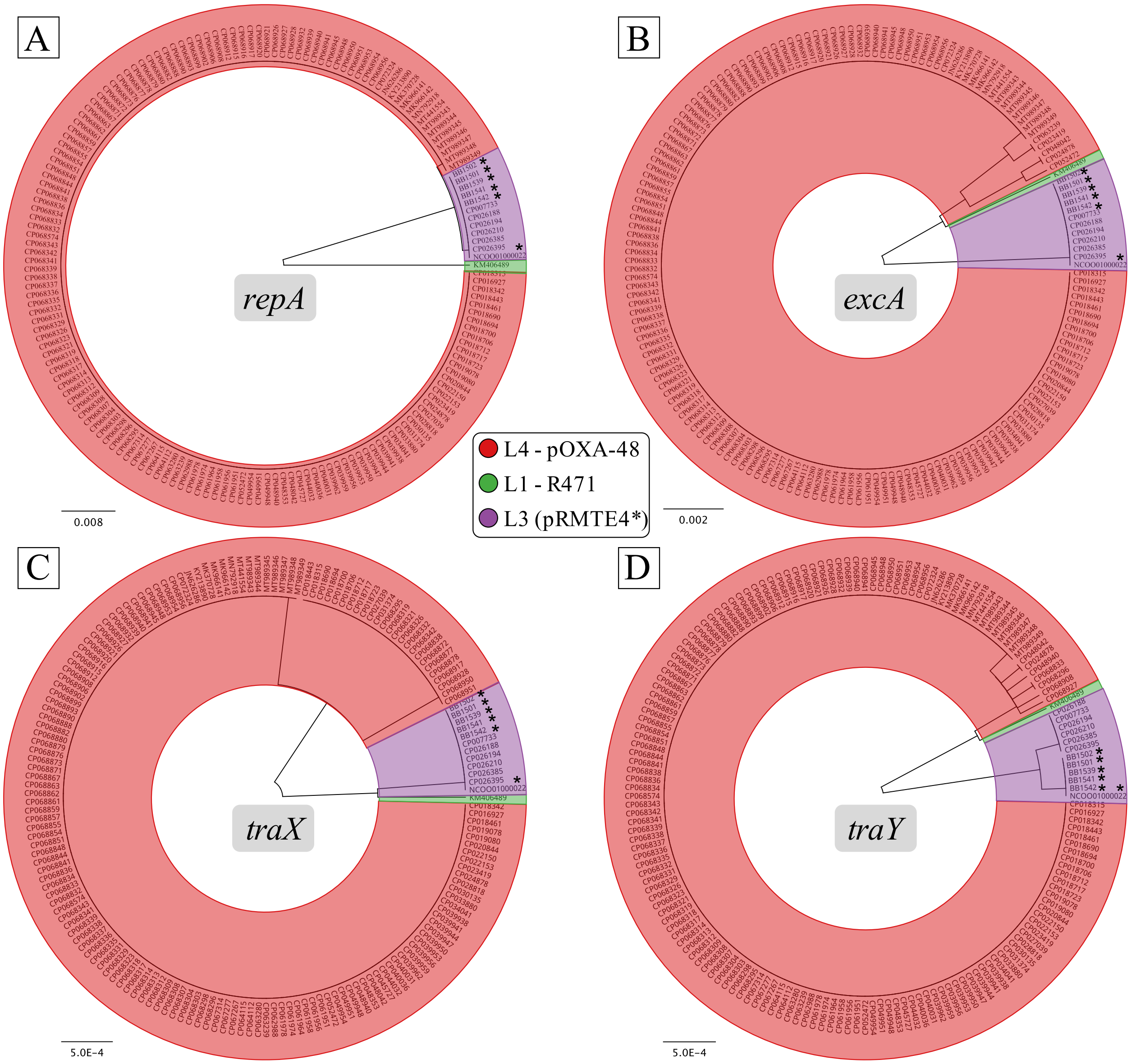

Supplementary Figure 2. SNP-trees of genes considered molecular markers for phylogenetic classification of IncL plasmids (scale bars: substitutions per site). Specific IncL types are indicated by colored shades (center legend) and pRMTE4 plasmids are pointed out by ‘*’. Substitution rate scale is shown in the bottom-left of each figure panel. A: *repA* SNP-tree. B: *excA* SNP-tree. C: *traX* SNP-tree. D: *traY* SNP-tree.

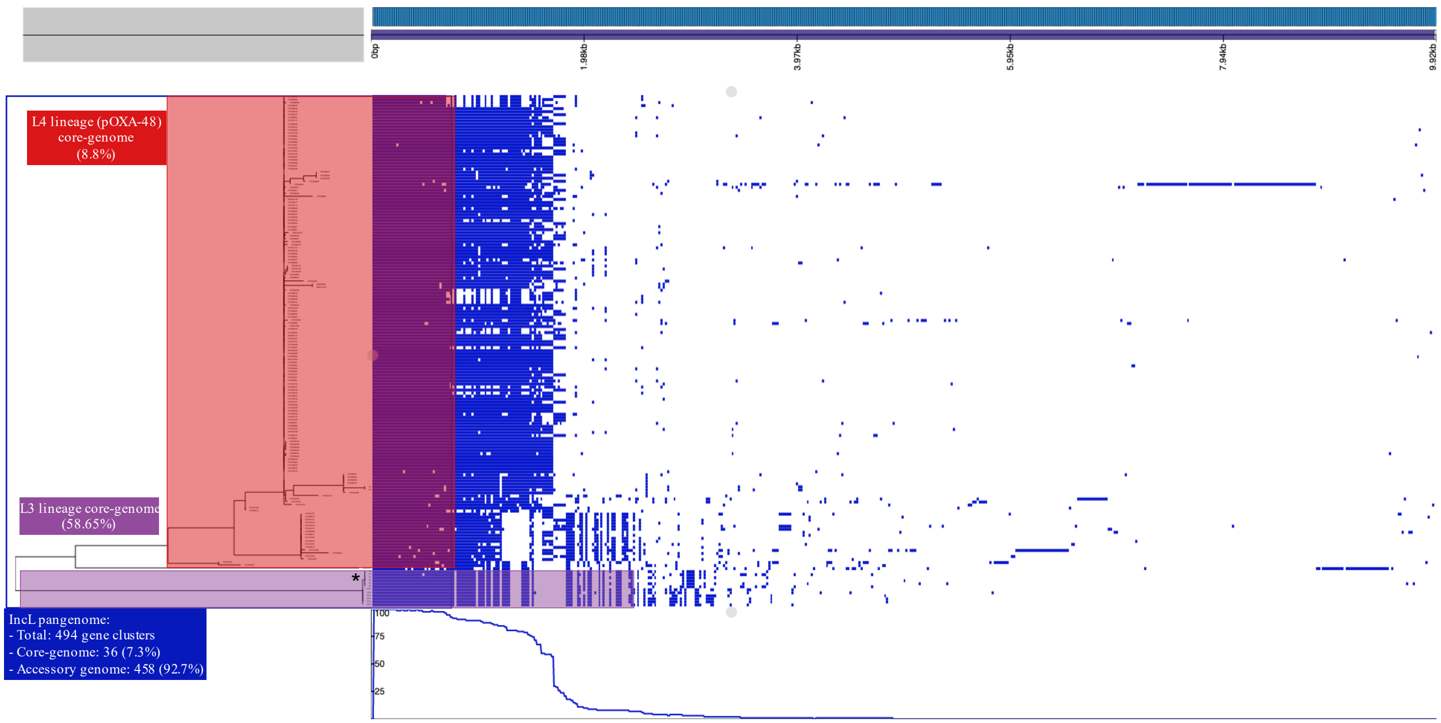

Supplementary Figure 3. Pan-genome visualization of all IncL type plasmids. Phylogenetic tree was constructed based on SNPs from non-recombinogenic core-genes present in at least 95% of 169 Incl plasmids included in the collection. The graph shows the presence (dark blue) and absence (white) in each plasmid of all gene clusters encompassed in the pan-genome. Total IncL core-genome and pOXA-48 cluster and L3 lineage core-genomes are highlighted by different colored frames, indicating in each case the % of gene clusters belonging to core-genome with respect to the total gene clusters for each IncL plasmid type. pRMTE4 plasmid cluster is pointed out by '*'.

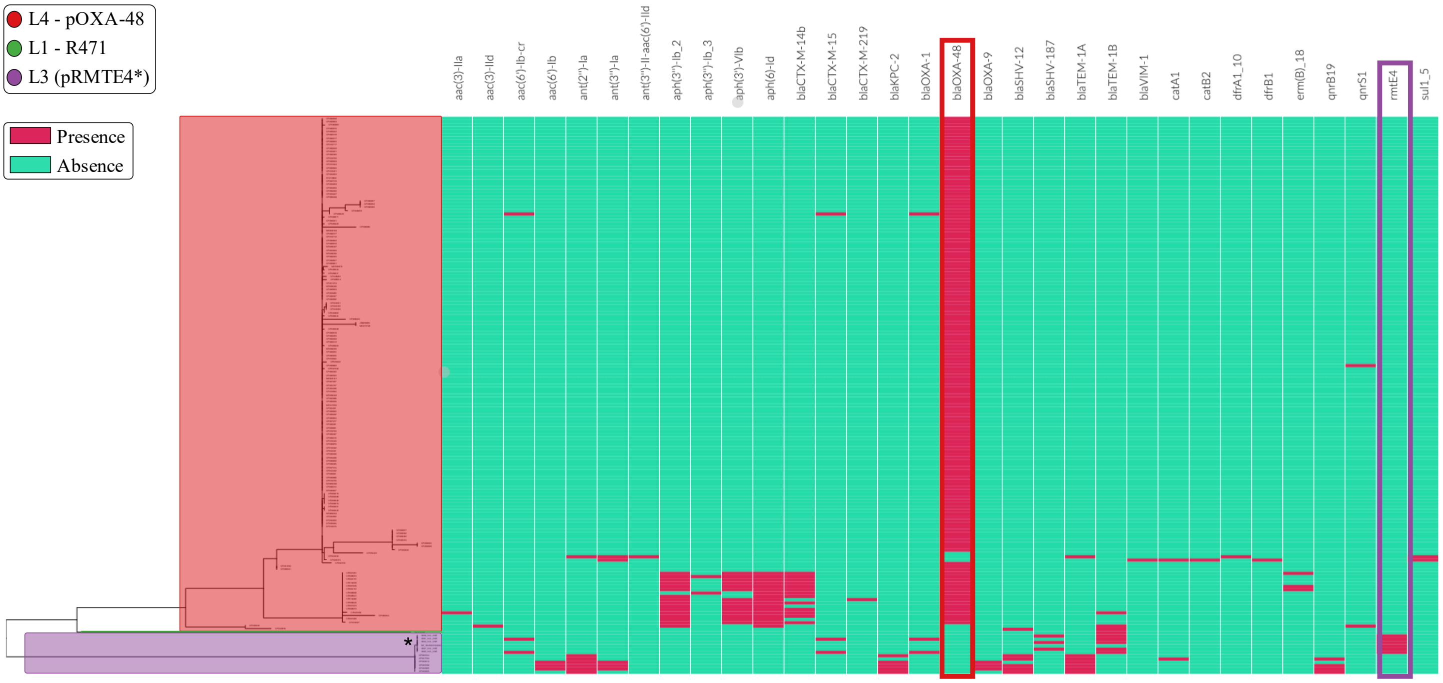

Supplementary Figure 4. Resistance gene content of all IncL type plasmids. Phylogenetic tree was constructed based on SNPs from non-recombinogenic core-genes present in at least 95% of 169 Incl plasmids included in the collection. Specific IncL types are indicated by colored shades (top-left legend) and pRMTE4 plasmid cluster is pointed out by '*'. The graph shows the presence and absence in each plasmid of all antimicrobial resistance genes identified in the IncL plasmid collection. Key resistance genes for pOXA-48 and pRMTE4 plasmids are emphasized by colored frames.
